# Evidence of a synthetic lethality interaction between SETDB1 histone methyltransferase and CHD4 chromatin remodeling protein in a triple negative breast cancer cell line

**DOI:** 10.1590/1414-431X2023e12854

**Published:** 2023-11-13

**Authors:** M.S. Moraes-Almeida, M.C. Sogayar, M.A.A. Demasi

**Affiliations:** 1 Centro de Terapia Celular e Molecular (NUCEL), Faculdade de Medicina, Universidade de São Paulo, São Paulo, SP, Brasil; 2 Departamento de Bioquímica, Instituto de Química, Universidade de São Paulo, São Paulo, SP, Brasil

**Keywords:** Breast cancer, Synthetic lethality, CRISPR-Cas9, CHD4 chromatin remodeling protein, SETDB1 histone methyltransferase

## Abstract

During the tumorigenic process, cancer cells may become overly dependent on the activity of backup cellular pathways for their survival, representing vulnerabilities that could be exploited as therapeutic targets. Certain molecular vulnerabilities manifest as a synthetic lethality relationship, and the identification and characterization of new synthetic lethal interactions may pave the way for the development of new therapeutic approaches for human cancer. Our goal was to investigate a possible synthetic lethal interaction between a member of the Chromodomain Helicase DNA binding proteins family (CHD4) and a member of the histone methyltransferases family (SETDB1) in the molecular context of a cell line (Hs578T) representing the triple negative breast cancer (TNBC), a subtype of breast cancer lacking validated molecular targets for treatment. Therefore, we employed the CRISPR-Cas9 gene editing tool to individually or simultaneously introduce indels in the genomic loci corresponding to the catalytic domains of SETDB1 and CHD4 in the Hs578T cell line. Our main findings included: a) introduction of indels in exon 22 of *SETDB1* sensitized Hs578T to the action of the genotoxic chemotherapy doxorubicin; b) by sequentially introducing indels in exon 22 of *SETDB1* and exon 23 of *CHD4* and tracking the percentage of the remaining wild-type sequences in the mixed cell populations generated, we obtained evidence of the existence of a synthetic lethality interaction between these genes. Considering the lack of molecular targets in TNBC, our findings provided valuable insights for development of new therapeutic approaches not only for TNBC but also for other cancer types.

## Introduction

During the tumorigenic process, cancer cells may become overly reliant on backup or alternative molecular processes for their survival, distinguishing them from their normal counterparts and constituting vulnerabilities that could be exploited as therapeutic targets (1). Certain vulnerabilities manifest as a synthetic lethality relationship between two genes or pathways, and therefore, application of the synthetic lethality concept is particularly promising for developing new therapeutic approaches for cancers harboring loss-of-function mutations in tumor suppressor genes (2).

One molecular process that has attracted interest over the last few years is the dynamic modification of the chromatin architecture (3). Loss-of-function and gain-of-function mutations promoting alterations in the activity of a specific chromatin modifier have the potential to perturb the transcriptional activity of several genes, constituting an important molecular mechanism associated to the disruption of crucial processes in tumorigenesis, such as cell proliferation and differentiation control and DNA repair (4). Few synthetic lethal relationships between histone-modifying enzymes and proteins that comprise chromatin remodeling complexes are known. Considering the intricate functional relationship between these two classes of protein groups, the existence of additional synthetic lethality interactions between these protein groups appears to be highly likely (5). In this work, we set out to investigate a possible synthetic lethality interaction between SETDB1 (SET Domain Bifurcated Histone Lysine Methyltransferase 1) and CHD4 (Chromodomain Helicase DNA-Binding Protein 4). The *SETDB1* is one of the methyltransferases that catalyse the addition of two or three methyl radicals to lysine 9 of histone H3 (H3K9me2 and H3K9me3, respectively). CHD4 has a dual function as part of the nucleosome remodeling and deacetylase (NuRD) complex, acting as a major ATPase subunit of this complex and recognizing modified histone tails, including H3K9me3, via its PHD domain (6). By employing CRISPR guide RNAs targeting the genomic regions encoding critical functional domains of SETDB1 and CHD4, we provide here the first evidence for the existence of a synthetic lethality interaction between the genes encoding these proteins.

## Material and Methods

The experimental procedures employed in this work were reviewed and approved by the Commission of Ethics in Research of the School of Medicine of University of São Paulo (CAAE 67573517.8.0000.0065).

### Cell culture

Hs578T (ATCC: HTB-126) and HEK-293T (provided by Prof. Eugenia Costanzi-Strauss, from the Department of Cell Biology, Institute of Biomedical Sciences, University of São Paulo) cell lines were cultured in D-MEM medium supplemented with 10% fetal bovine serum (Cultilab, Brazil).

### Gene expression analysis by reverse transcription followed by polymerase chain reaction (RT-PCR)

To evaluate mRNA expression of the *SETDB1* and *CHD4* genes in the Hs578T cell line, total RNA was purified employing the RNAspin Mini RNA isolation kit (Cytiva, formerly GE Healthcare, USA), following the manufacturer's guidelines. cDNA was synthesized from total RNA samples according to the manufacturer's instructions (Maxima H Minus Transcriptase, ThermoScientific, USA), and PCRs were carried out under standard reaction conditions using the primers shown in Table 1.

**Table 1:** Primers used for gene expression analysis by RT-PCR.

Gene	Sequence
*CHD4* (Forward)	5′ ACGCATCGATGGTGGAATC 3′
*CHD4* (Reverse)	5′ ATGGGGGTTCCAGTCAGAGT 3′
*SETDB1* (Forward)	5′ CGGCTACAGCTATTCAAGACACAGA 3′
*SETDB1* (Reverse)	5′ TACTCATCACCCATTTCCAGACCC 3′
*GAPDH* (Forward)	5′ TGCACCACCAACTGCTTAGC 3′
*GAPDH* (Reverse)	5′ GGCATGGACTGTGGTCATGAG 3′

### Design of the single guide RNAs (sgRNA) and expression vectors

All sgRNAs (synthesized by Life Technologies/ThermoFischer, Brazil) used target functional domains of SETDB1 and CHD4 and displayed a MIT Specificity score >70 to minimize off-target events; potential off-targets presented at least three mismatches. As a non-target control, we used the 5M sgRNA specific for the murine *Chd7* gene (synthesized by Life Technologies/ThermoFischer), presenting two mismatches to the human gene (Table 2).

**Table 2:** Specific sgRNA and MIT Specificity score.

Gene	Sequence	MIT Specificity score
*CHD4(1)*	5′ CACCGTCATCCGAGAGAATGAGTTC 3′	92
*CHD4(2)*	5′ CACCGTGGGGGCCTTGGAATCAATC 3′	84
*SETDB1(1)*	5′ CACCGAACCTGTTTGTCCAGAATGT 3′	73
*SETDB1(2)*	5′ CACCGTGGGACTACAACTACGAGGT 3′	93
*5M*	5′ CACCGGTAATATTCCTTGGCGCTGT 3′	-

The sgRNAs oligos were cloned into BsmB1-digested pL-CRISPR.EFS.GFP (Addgene #57818) and pL-CRISPR.V2 (Addgene #104994) third generation lentiviral expression vectors (7). Addgene (https://www.addgene.org/) is a non-profit plasmid repository.

### Production of recombinant lentiviral particles and transduction of Hs578T cells

HEK-293T cells were transfected with the transfer vectors obtained and the helper expression plasmids pHDM-HGPM2, pREV, pTAT, and pVSVG by lipofection (Lipofectamine 2000, Invitrogen, USA). For transduction of the Hs578T target cell line, the cells were submitted to the spinfection protocol (8) at a multiplicity of infection of 10. The mixed cell populations obtained were subjected to either FACS enrichment for EGFP^+^ cells (pL-CRISPR.EFS.GFP) or selection with puromycin (2 µg/mL) (pLenti-CRISPR.V2).

### Confirmation of gene editing by TIDE (Tracking of Indels by Decomposition)

The target genomic sites were amplified by PCR employing 35 ng of gDNA and the primers are shown in Table 3.

**Table 3:** Primers employed in the amplification of the *CHD4*, *SETDB1*, and *5M* genomic target sites.

Gene	Sequence
*CHD4(1)* (Forward)	5′ GGGGAGGAGGGGAGTTCCTTGCCA 3′
*CHD4(1)* (Reverse)	5′ TGGGGGCTCCAACATCCCTCCCT 3′
*CHD4(2)* (Forward)	5′ TGTGCCACCACGGGCCCAGATT 3′
*CHD4(2)* (Reverse)	5′ GCACCCCTGCCTCCAGACAC 3′
*SETDB1(1)* (Forward)	5′ GGGACCAGCCGAAAGCCCACTGC 3′
*SETDB1(1)* (Reverse)	5′ TGGGAGGGATGTCAGGCCGAGGT 3′
*SETDB1(2)* (Forward)	5′ AGGGGGAAAGTGGGACCAGCCGA 3′
*SETDB1(2)* (Reverse)	5′ GATCCTGCCTGCTAGCACCAC 3′
*5M* (Forward)	5′ GAATTCCGAACCTGGACAGA 3′
*5M* (Reverse)	5′ GTAAGAATCCAAAAGGCAAC 3′

The purified PCR fragments (100 ng) were sequenced (Big Dye Terminator kit, Applied Biosystems, USA), using the primers shown in Table 4.

**Table 4:** Sequencing primers.

Gene	Sequence
*CHD4(1)*	5′ GATGCCTCTTTCTGCACATG 3′
*CHD4(2)*	5′ GCACCCCTGCCTCCAGACAC 3′
*SETDB1(1)*	5′ CAAGTAAGTTCTGTCCCAGC 3′
*SETDB1(2)*	5′ GATCCTGCCTGCTAGCACCAC 3′
*5M*	5′ GTAAGAATCCAAAAGGCAAC 3′

Sanger chromatogram traces were analyzed with the TIDE web tool using the default parameters (9,10).

### T7 endonuclease assay

Target DNA fragments were PCR-amplified as described above, using SETDB1 forward and reverse primers (2). The 1:1 mixture of PCR amplicons were prepared and processed according to manufacturer instructions (New England Biolabs, USA). The estimated percentages of events involving DNA cutting, followed by non-homologous end-joining, were calculated as described previously (11).

### Determination of population doubling time

The number of cells 48 and 72 h after plating was determined, and the doubling times were estimated using the following equation:

Doubling time = ln(N_72h_N_48h_) / (Δt × ln2), where N_72h_ and N_48h_ are the cell numbers after 72 and 48 h, respectively.

### Evaluation of doxorubicin cytotoxicity

A dose-response curve to the chemotherapeutic drug Doxorubicin (DOX, Sigma, USA) was generated. After treatment for 72 h, the cultures were submitted to the MTT cell viability assay. The absorbance values associated to each treatment point are reported as percentage of inhibition (%Inhibition) relative to that associated to untreated control cells as follows: %Inhibition = 100 × [1 - (ABS_t_ - ABS_min_) / (ABS_max_ - ABS_min_)], where ABS_t_ is the absorbance associated to treatment with a determined DOX dose, ABS_min_ is the absorbance value when DOX concentration is maximal (2.25 µM), and ABS_max_ is the absorbance when DOX concentration is 0.

Half maximal inhibitory concentration (IC_50_) values were obtained by fitting the %Inhibition *vs* DOX concentration using the logistic model.

## Results

### Evaluation of *CHD4* and *SETDB1* transcriptional activity in the Hs578T breast cancer cell line

**Figure 1:**
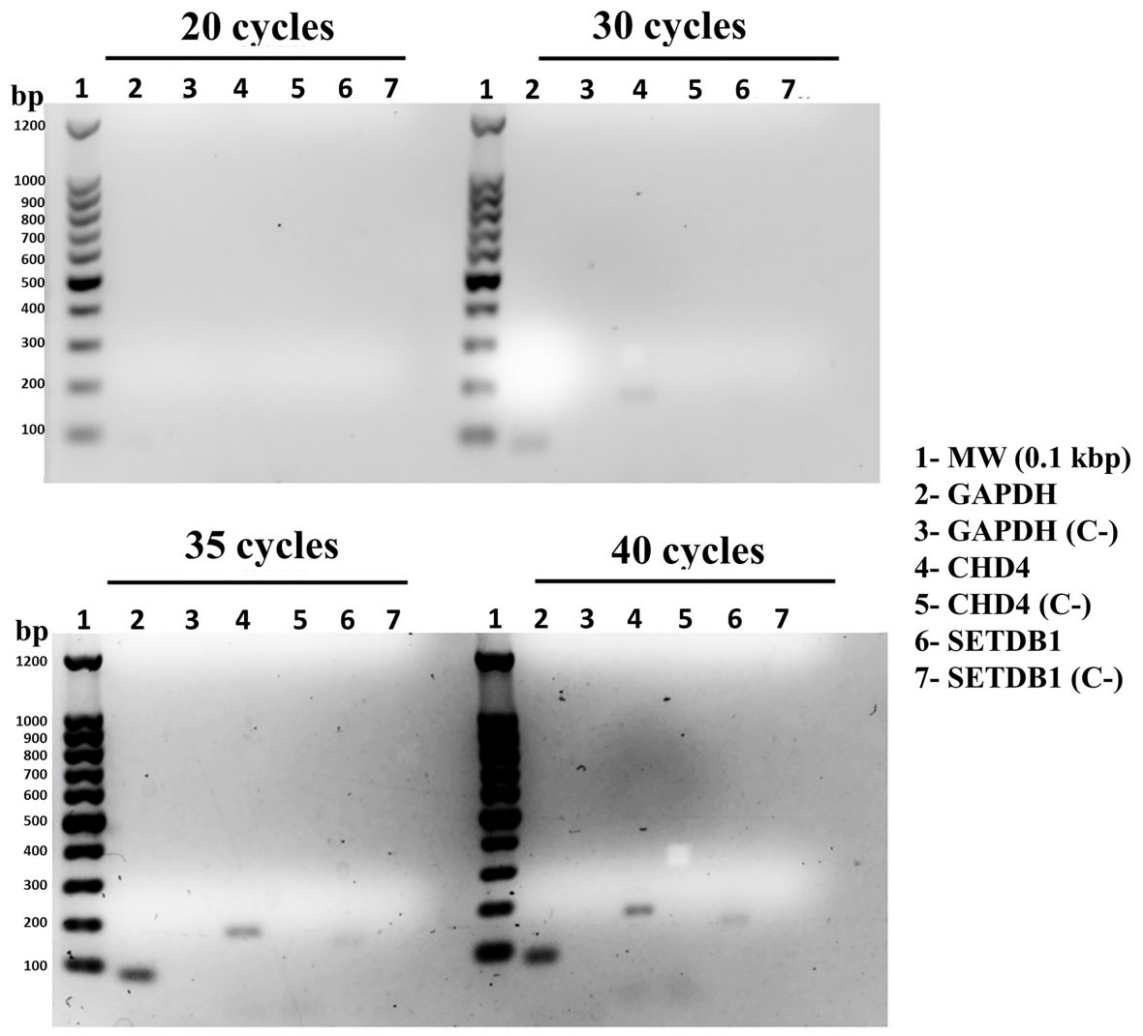
Evaluation of the expression of the *CHD4* and *SETDB1* genes in the Hs578T human triple negative breast cancer cell line by RT-PCR. cDNA samples were used to detect the mRNAs of the *CHD4* and *SETDB1* genes using specific primers for each gene analyzed. Expression of the *GAPDH* gene mRNA was used as an endogenous control. For each primer pair and PCR run, a no-template-control (without cDNA) was included. Amplification of specific DNA fragments was evaluated after 20, 30, 35, and 40 PCR cycles, as indicated. The expected sizes of the amplified DNA fragments are 86, 171, and 148 bp for the *GAPDH*, *CHD4*, and *SETDB1* mRNAs, respectively. Fractionation was performed through 1% agarose gel electrophoresis.

The transcriptional activity of the *CHD4* and *SETDB1* genes was evaluated by RT-PCR. Figure 1 shows the mRNA amplicons of a region of *GAPDH*, a ubiquitously expressed gene used as a control in the experiment, and *CHD4* and *SETDB1* genes, confirming that the *CHD4* and *SETDB1* genes are transcriptionally active in the Hs578T cell line.

### Individual gene editing of *SETDB1* and *CHD4* genes in Hs578T cells

**Figure 2:**
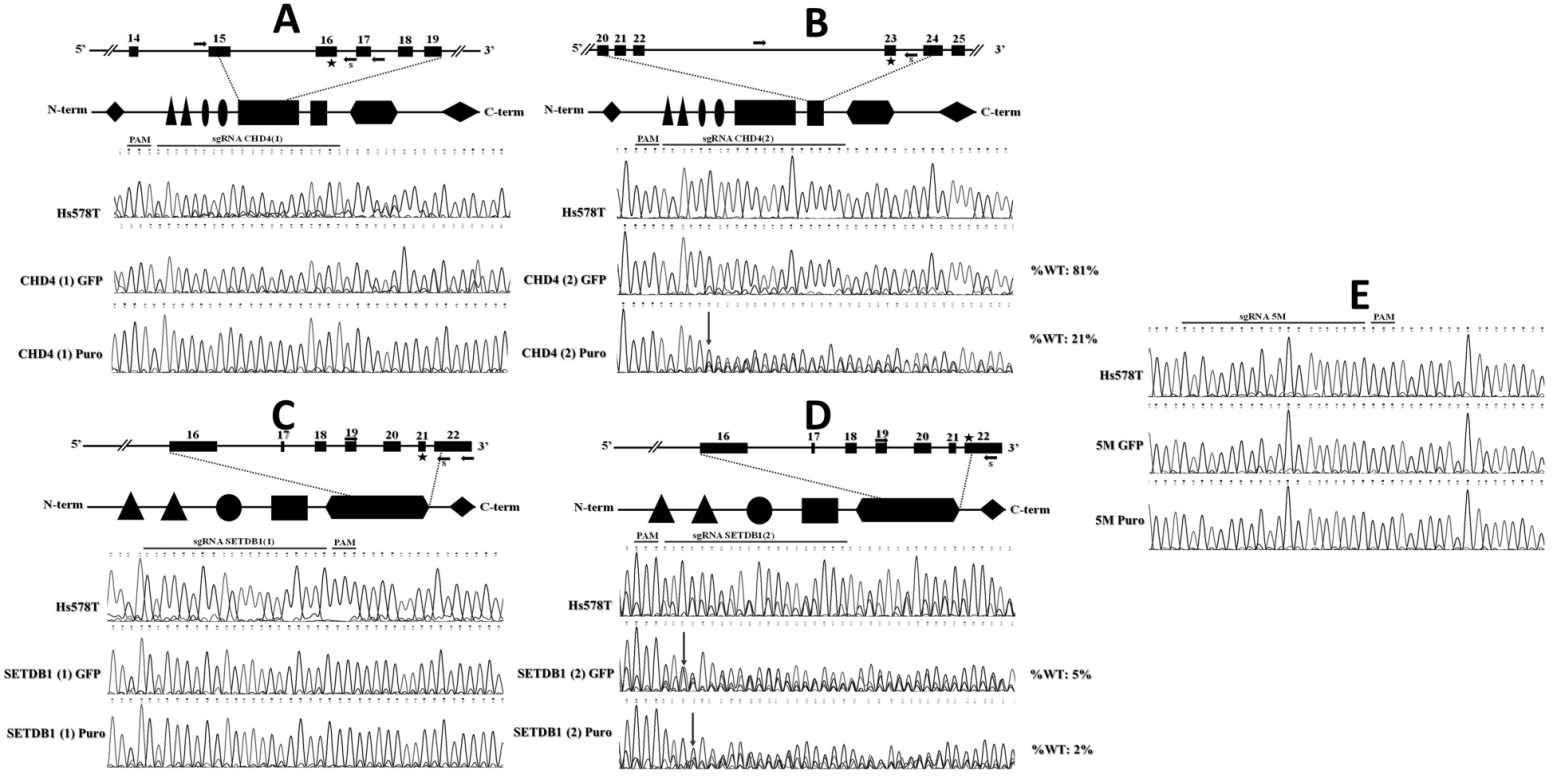
Validation of disruption of *CHD4* and *SETDB1* genes by TIDE-coupled Sanger sequencing. Representative chromatograms, obtained by Sanger sequencing, of the genomic target regions from Hs587T cells and the indicated mixed cell populations associated to sgRNAs CHD4(1) (**A**), CHD4(2) (**B**), SETDB1(1) (**C**), SETDB1(2) (**D**), and the non-target control sgRNA 5M (**E**). Above each set of chromatograms associated to *CHD4* and *SETDB1* genes is a diagram representing the genomic region targeted by the specific sgRNA. The exons are numbered and represented as boxes, the genomic region targeted by the sgRNA is indicated by a star, and the primers used to amplify the genomic target region are indicated by arrows. The sequencing primer is indicated with the letter “S”. Below the representation of the genomic regions are illustrations of the respective protein domains, highlighting the relationship between the protein domain affected by the genomic disruption and the corresponding exons coding for these particular domains. The indel mutations caused by the ectopic expression of sgRNAs CHD4(2) and SETDB1(2) are indicated as downward arrows in the chromatograms. %WT: percentage of the remaining wild-type genomic sequence, as determined by TIDE analysis. CHD4 domains legend: diamond: chromodomain helicase DNA binding; triangle: plant homeodomain; ellipse: chromodomain; rectangle: SNF2/Helicase C; trapezium: domain of unknown function. SETDB1 domain legend: triangle: tudor domains; ellipse: methyl-CpG-binding domain; rectangle: pre-SET domain; trapezium: SET domain; diamond: post-SET domain.

We employed sgRNAs targeting exons 21 and 22 of *SETDB1* (denoted SETDB1(1) and (2), respectively, corresponding to the SET methyltransferase catalytic domain) and exons 16 and 23 of *CHD4* (denoted CHD4(1) and (2), respectively, corresponding to the ATP-dependent helicase domain). Disruption of *SETDB1* and *CHD4* genes was evaluated by TIDE-coupled Sanger sequencing analysis (Figure 2).

Our results indicated that only sgRNAs SETDB1(2) and CHD4(2) were effective in creating indels at the expected genomic sites (Figure 2A-D). No indels were found to be associated to expression of the 5M sgRNA targeting exon 13 of the *CHD7* gene, used as non-target control in both mixed cell populations evaluated (5M GFP and 5M Puro; Figure 2E). Based on these results, we selected the mixed cell populations SETDB1(2)GFP, CHD4(2)Puro, 5M GFP, and 5M Puro for further analysis.

### Impact of individual gene editing of *SETDB*1 and *CHD4* on growth rate and chemosensitivity to DOX of Hs578T cells

**Figure 3:**
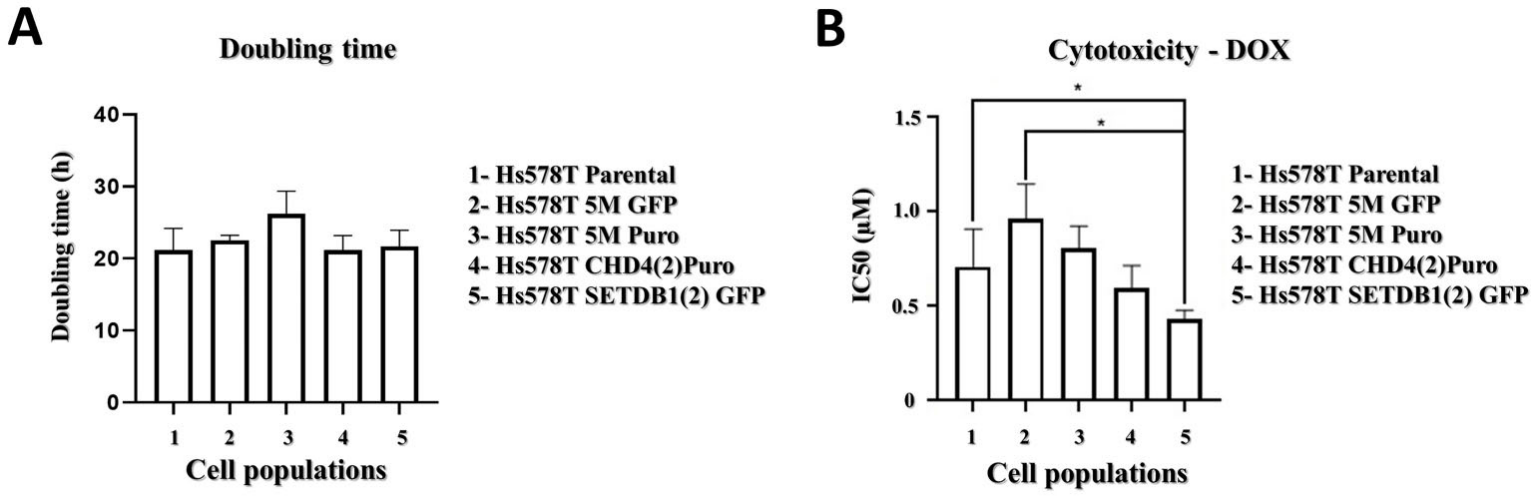
Analysis of the growth rate and chemosensitivity to DOX of Hs578T cells associated to individual gene editing of *SETDB1* and *CHD4.* **A**, Histogram representing the average (SD) doubling time values of parental Hs578T cells and of the indicated mixed cell populations. Experiments were carried out in triplicate. **B**, Histogram illustrating the average (SD) half maximal inhibitory concentration (IC_50_) values for DOX displayed by the parental Hs578T cells and by the indicated mixed cell populations. *P<0.05, ANOVA, followed by Tukey’s test.

The estimated average doubling time for Hs578T parental cells was approximately 23 h (Figure 3A), similar to other doubling time estimates for Hs578T cells published in the literature (8,12-14). Apparently, indel events in exon 23 of the *CHD4* gene and exon 22 of the *SETDB1* gene are not related to changes in the proliferative capacity of Hs578T cells (Figure 3A).

Next, we set out to investigate whether gene editing of *SETDB1* or *CHD4* could sensitize Hs578T cells to DOX (Figure 3B), a genotoxic agent (15).

The average IC_50_ for DOX in Hs578T cells (0.70±0.2 µM) was within the IC_50_ range for DOX in Hs578T cells described in the literature (16-18). Only the mixed cell population SETDB1(2)GFP presented an average IC_50_ for DOX (0.43±0.05 μM) that was statistically lower than the average for Hs578T and 5M GFP (0.96±0.19 µM) cells (Figure 3B). This result suggested that the indel events promoted in exon 22 of the *SETDB1* gene sensitized Hs578T cells to the cytotoxic action of DOX.

### Evidence for a synthetic lethality interaction between *SETDB1* and *CHD4* in Hs578T cells

**Figure 4:**
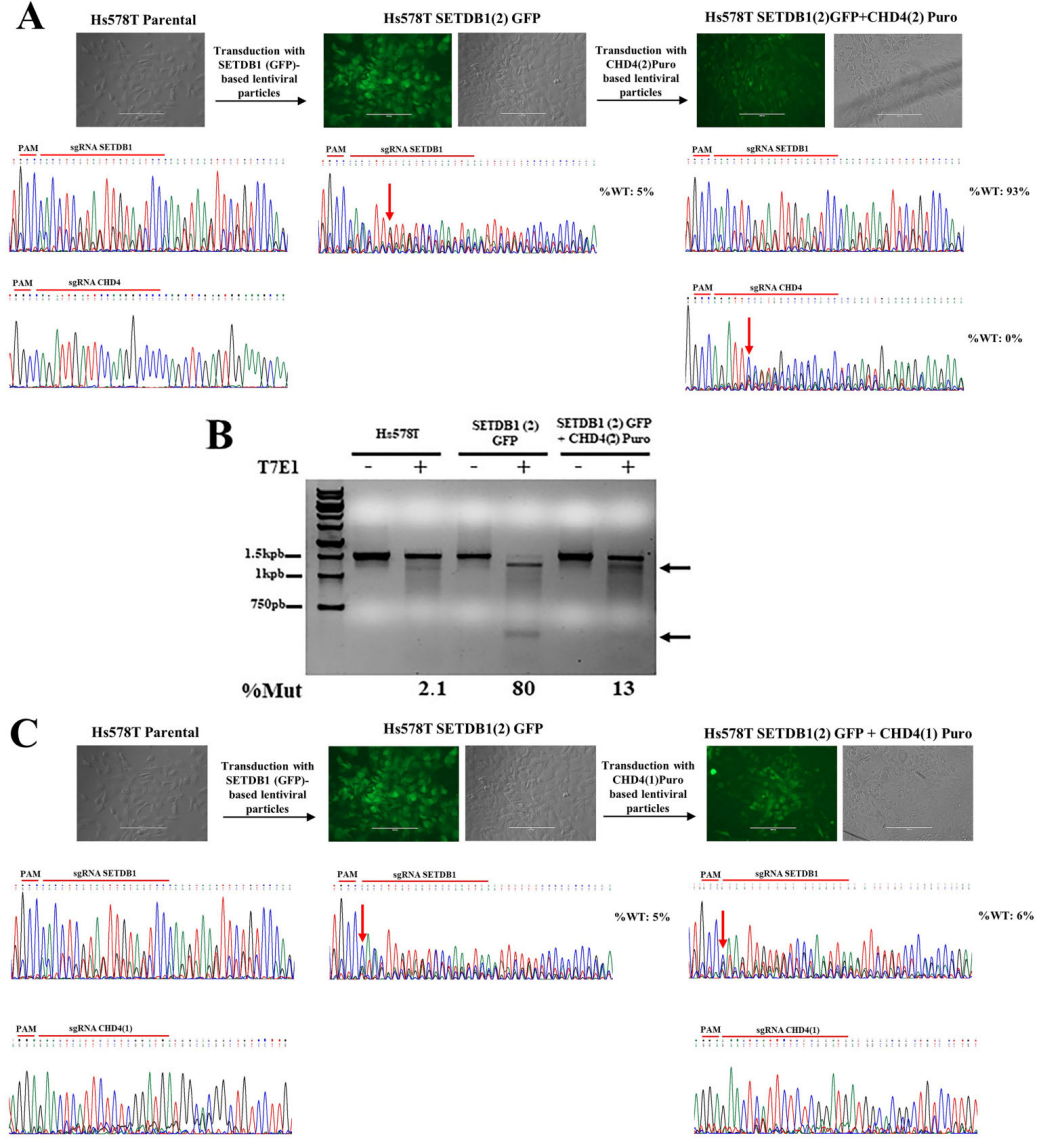
Evidence for *CHD4* and *SETDB1* synthetic lethal interaction by tracking their simultaneous disruption. **A**, The upper part of the figure shows the experimental setup used to generate the mixed cell population stably expressing the sgRNAs for both SETDB1 and CHD4. Representative photomicrographs of confluent monolayers of parental Hs578T cells (phase contrast), SETDB1(2)GFP cells (phase contrast/fluorescence microscopy), and SETDB1(2)GFP+CHD4(2)Puro cells (phase contrast/fluorescence microscopy) are shown (scale bar 200 µm). The lower part shows the chromatograms obtained by Sanger sequencing of the genomic target regions from Hs587T cells and mixed cell populations SETDB1(2)GFP and SETDB1(2)GFP+CHD4(2)Puro. The indel mutations caused by the ectopic expression of sgRNAs *CHD4* and *SETDB1* are indicated by downward arrows in the chromatograms. %WT: percentage of the remaining wild-type genomic sequence, as determined by TIDE analysis. **B**, Representative 1% agarose gel electrophoresis image of the T7E1 assay carried out with PCR products associated to the *SETDB1* genomic target site and amplified from gDNA obtained from the indicated cell populations. %Mut: estimated frequencies of mutational events at exon 22 of *SETDB1*. **C**, Same experiment as described in (**A**), but carried out with the CHD4(1)Puro (not functional) sgRNA instead of the CHD4(2) sgRNA (scale bar 200 µm).

Next, we evaluated the effect of the simultaneous gene editing of *SETDB1* and *CHD4* genes on the proliferative potential of Hs578T cells employing the mixed cell population SETDB1(2)GFP as a starting point due to the low frequency (∼5%) of the remaining wild-type sequence of exon 22 of *SETDB1* in this cell population (Figure 2D). The SETDB1(2)GFP mixed cell population was transduced with the pL-CRISPR.V2.CHD4 lentiviral vector, followed by selection with puromycin, generating the mixed cell population named SETDB1(2)+CHD4(2)Puro. Simultaneous disruption of *SETDB1* and *CHD4* genes was evaluated by TIDE-coupled Sanger sequencing analysis (Figure 4).

As shown in Figure 4A, after establishment of the SETDB1(2)+CHD4(2)Puro mixed cell population, the frequency of the remaining wild-type sequence of exon 22 of *SETDB1* dramatically increased from ∼5 to ∼93%. This result was confirmed by the T7 endonuclease assay (Figure 4B). Disruption of exon 23 of *CHD4* was effective in the SETDB1(2)+CHD4(2)Puro mixed cell population (Figure 4A). Conversely, the frequency of the remaining wild-type sequence of *SETDB1* exon 22 remained practically stable when non-effective sgRNA CHD4(1) was expressed in SETDB1(2)GFP cells (Figure 4C). Altogether, these results indicated that upon puromycin selection, most of the fluorescent cells composing the SETDB1(2)GFP+CHD4(2)Puro mixed cell population contained only editions at exon 23 of *CHD4*, indicating an enrichment for fluorescent cells not originally edited at exon 22 of *SETDB1* in the SETDB1(2)GFP population. Therefore, our results suggested that the simultaneous disruption of exon 22 of *SETDB1* and exon 23 of *CHD4* is either lethal or associated with impaired cell proliferation potential, indicating a synthetic lethality interaction between *SETDB1* and *CHD4* genes in the molecular context of Hs578T cells.

## Discussion

The two genes evaluated in the present study are classified as “common essential genes” by the Cancer Dependency Map (19). Regarding the Hs578T cell line, *CHD4* and *SETDB1* display CERES dependency scores of -1.18 and -0.62, respectively, indicating that these genes, particularly *CHD4,* have a high likelihood of being essential to this cell line. However, we were not able to detect a clear effect of the introduction of indels in exon 23 of *CHD4* or exon 22 of *SETDB1* in the proliferation potential of Hs578T (Figure 3A). One reason for this discrepancy may be related to differences in the observational period, since in our study the doubling time estimate was obtained after 72 h of cultivation, while the CERES dependency score is associated to a 21-day cultivation period (20).

The gene editing events at *SETDB1* exon 22 sensitized Hs578T cells to DOX treatment (Figure 3B). The cytotoxicity related to DOX exposure involves induction of DNA double-strand breaks associated to its intercalation into DNA, inhibition of Topoisomerase II, and generation of free radicals (15). Hs578T cells are relatively resistant to DOX-induced cytotoxicity (21), and numerous resistance mechanisms to DOX have been proposed (22), including epigenetic reprograming (23). Interestingly, Al Emran et al. (24) recently reported a correlation between gain of H3K9me3 marks associated to upregulation of histone methyltransferases *SETDB1* and *SETDB2* to induction of a DOX-tolerant phenotype in some cancer cell lines, including the SKBR3 breast cancer cell line, suggesting that a similar mechanism could be operating in Hs578T cells.

To our knowledge, we are providing the first experimental evidence for a synthetic lethal interaction between *CHD4* and *SETDB1*. Only two descriptions are available in the literature on synthetic lethal interactions involving either *CHD4* or *SETDB1*. A synthetic lethal interaction between *CHD4* and *PARP1* (Poly(ADP-ribose) polymerase-1) has been described in which CHD4 deficiency impairs the homologous recombination mechanism of DNA double-strand breaks, sensitizing CHD4-depleted cells to PARP1 inhibitors (25). Regarding *SETDB1*, a synthetic lethal interaction has recently been described in *Caenorhabditis elegans* between its MET-2 and BRCA-1 orthologs. Interestingly, the transcriptional silencing associated to H3K9me2 and H3K9me3 marks is also associated to the transient formation of repressive chromatin, apparently an initial and crucial step in activation of the repair process of DNA double strand breaks (DSBs) (26). Since SETDB1 is one of the methyltransferases responsible for introducing H3K9me2 and H3K9me3 marks and considering the ability of CHD4 to recognize H3K9me3 marks and the role of CHD4-containing NuRD complexes in DSBs repair process (27), it is tempting to speculate on a possible mechanistic link between these two genes involving the maintenance of genomic stability, and possibly suggesting a specific vulnerability of Hs578T cells.

More work is needed to characterize the synthetic lethal interaction between *CHD4* and *SETDB1* in TNBC and other cancers. In this line, it would be important to evaluate this synthetic lethality interaction in normal cell lines and other cancer cell models. It would be also valuable to evaluate whether *SETDB1* inhibition would sensitize other cell lines that are resistant to DOX-induced cytotoxicity, especially in cancer cell lines harboring *SETDB1* amplification.
